# Supplementary material for: The Phytochemical Analysis of Vinca L. Species Leaf Extracts Is Correlated with the Antioxidant, Antibacterial, and Antitumor Effects
Source: Molecules. 2021 May 19;26(10):3040. doi: 10.3390/molecules26103040 (PMC8160922; doi:10.3390/molecules26103040)
Supplement: Supplementary file 1 [file molecules-26-03040-s001.zip › molecules-1220158-supplementary.pdf]

molecules

Supplementary Materials

The Phytochemical Analysis of *Vinca* L. Species Leaf Extracts Is Correlated with the Antioxidant, Antibacterial, and Antitumor Effects

Alexandra Ciorîță [1,2,*], Cezara Zăgrean-Tuza [3], Augustin C. Moț [3], Rahela Carpa [1] and Marcel Pârvu [1]

[1] Faculty of Biology and Geology, Babeș-Bolyai University, 44 Republicii St., 400015 Cluj-Napoca, Romania; rahela.carpa@ubbcluj.ro (R.C.); marcel.parvu@ubbcluj.ro (M.P.)
[2] National Institute for Research and Development of Isotopic and Molecular Technologies, 67-103 Donath St., 400293 Cluj-Napoca, Romania
[3] Faculty of Chemistry and Chemical Engineering, Babeș-Bolyai University, 11 Arany János St., 400028 Cluj-Napoca, Romania; cezarazt7@gmail.com (C.Z.-T.); augustin.mot@ubbcluj.ro (A.C.M.)
* Correspondence: alexandra.ciorita@itim-cj.ro

Supplementary Methods

Antioxidant activities

- ABTS (REAC method)

The ABTS anionic radical was prepared by treating 5 mM reduced ABTS with 50 nM zucchini peroxidase and 1.3 mM hydrogen peroxide in 50 mM sodium acetate buffer of 5.5 pH. Further, 290 μL of the radical solution were pipetted along with 10 μL of each extract in a 96-well plate. The reaction mixture was incubated for 30 min and the absorbance was recorded at 730 nm afterwards, with bleaching percentages calculated as previously described. Rutin (in 3–23 μg/mL range) was used to obtain the calibration.

- CUPRAC

Neocuproine of 2 mM concentration, 1 mM pentahydrated $CuSO_4$, and 1 M ammonium acetate in pH 7, were incubated for 10 min. Then, 190 μL of this solution was mixed with 100 μL ultrapure water and 10 μL of each extract in a 96-well plate. After an additional 10 min of incubation, the 450 nm absorbance was recorded. A calibration curve with rutin (in 3–14 μg/mL range) was obtained.

All reagents were acquired from Sigma-Aldrich and the absorbances were recorded using a Tecan Spark multiplate reader.

Cytotoxicity assays

- MTT

After 48 h of incubation with the plant extracts, mitochondrial activity was assessed by MTT method. 3-(4,5-Dimethyl-thiazol-2-yl)-2,5Diphenyltetrazolium Bromide solution (100 μL) was added to each well in a final concentration of 0.5 mg/mL and left for incubation at 37 °C and 5% CO_2 for an additional 1.5 h. The resulted formazan was diluted in acidified iso-propanol and the absorbance was measured at 550 nm.

- LDH

50 μL of culture media was subtracted from test plates and together with 50 μL lithium lactate solution of 50 mM, 50 μL tris solution of 200 mM, and 50 μL of NAD solution (a mixture of ionitrotetrazolium violet, phenazine metosulphate, and nicotinamide dinucleotide) were added to a new 96 wells plate. The absorbance was read after 10 min at 490 nm and the background at 690 nm. The plate included also vehicle controls with media and each one of the extracts treated with LDH solution for the same amount of time to exclude possible errors.

- NO

Determination of nitric oxide (NO) concentration was done by Griess reaction. 50 μL of culture media was mixed with 50 μL sulphanilamide and the solution was kept in dark at room temperature for 10 min. *N*-(-1-napthyl)-ethylenediamine (N1-NAP) was added and the reaction was kept in dark at room temperature for 10 min. The samples were read at 540 nm.

All reagents used for cell toxicity assays were from Sigma-Aldrich (Germany), and the absorbances were read using BioTech Epoch plate reader and Gen5 software.

Ultrastructural analyses

The ultrastructural modifications induced by the extracts were examined through transmission electron microscopy (TEM). The samples were incubated on glass slides at a confluence of 3×10^4 cells/glass slide. The extracts were incubated with the cells at the median inhibitory concentration (IC_{50}) value determined through MTT assay, for 24 h, at 37 °C and 5% CO_2. After 24 h of incubation, the cells were fixed with 2.7 glutaraldehyde in 0.1 M phosphate buffer saline (PBS), for 1.5 h at 4 °C. After this, the slides were washed with PBS 4 times, one hour each, and post-fixed with osmium tetroxide for an additional hour. The samples were then washed four times with PBS (15 min each) and dehydrated with acetone in increasing concentrations (from 30% to 100%, each step for 15 min, at 4 °C). The glass slides were included in Epoxy resin Epon 812, upside down, so that the cells could be emerged in the resin. The infiltration took place for four hours (1:2, 1:1, 2:1, 1:0 with acetone) and the samples were then polymerized for 72 h at 60 °C. The slides were removed from the epoxy resin through thermic shock, by immersing the blocks in liquid nitrogen for two seconds. The ultrathin sections were obtained using Leica UC7 ultramicrotome (Leica, Germany), and the double stained sections (with uranyl acetate and lead citrate) were examined with TEM Jeol JEM 1010 (Jeol, Japan).

Supplementary Figures and Tables

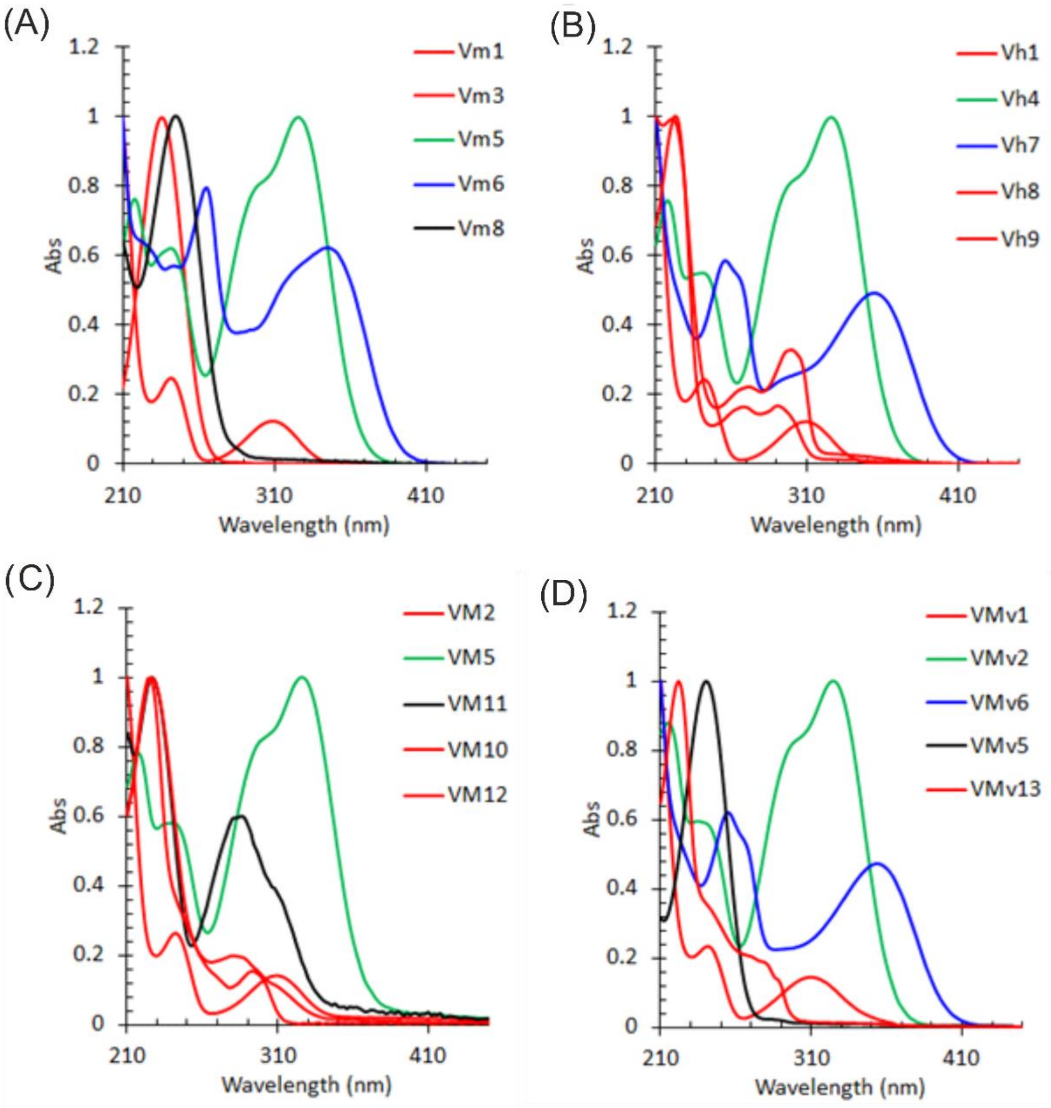

Figure S1. DAD UV-Vis molecular absorption spectra of representative compounds in the analyzed plant species extracts after chromatographic separation: (**A**) *Vinca minor* (Vm), (**B**) *Vinca herbacea* (Vh), (**C**) *Vinca major* (VM), (**D**) *Vinca major* var. *variegata* (VMv).

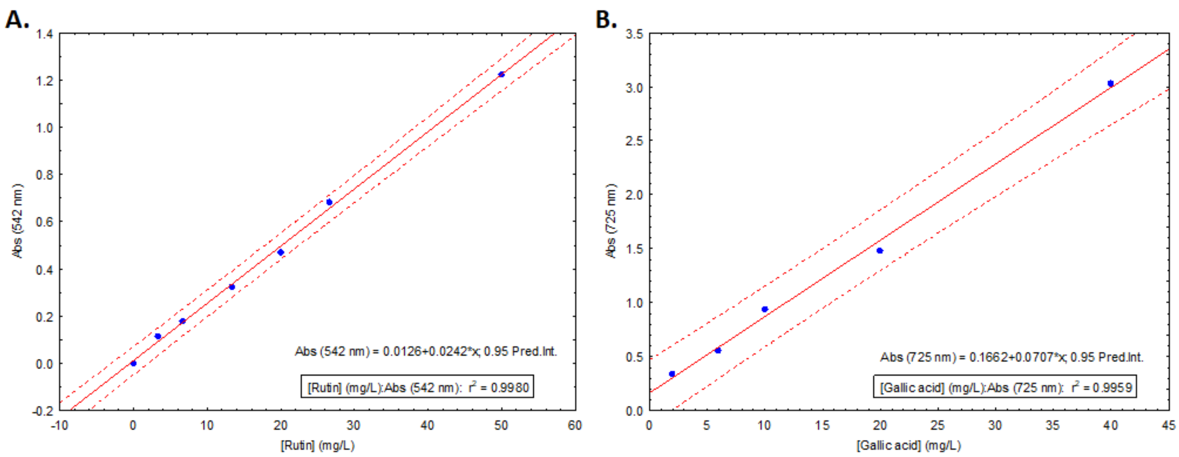

Figure S2. Calibration curves for rutin (TFC) and gallic acid (TPC).

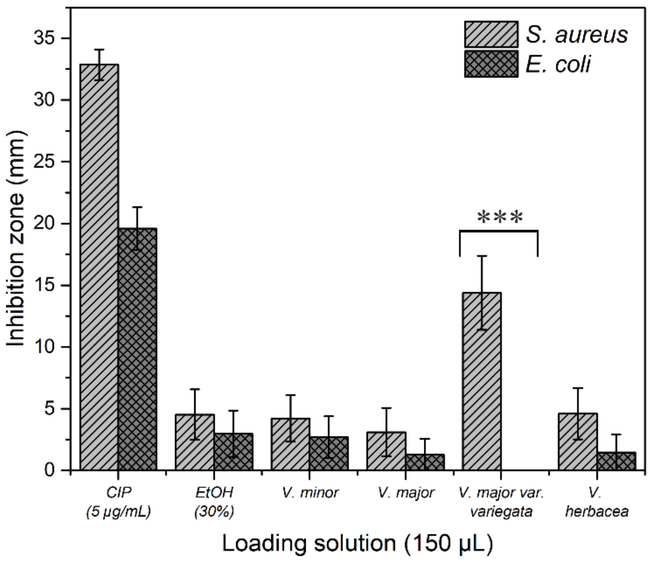

Figure S3. Graphical distribution of antibacterial activity assessed through agar well diffusion method. The values represent the mean of six individual experiments ±standard error of the mean; CIP = ciprofloxacin, EtOH = ethanol; Student's t test was performed to determine if the Gram-positive strain, *S. aureus*, is affected differently from the Gram-negative strain, *E. coli*; *** $p < 0.0001$.

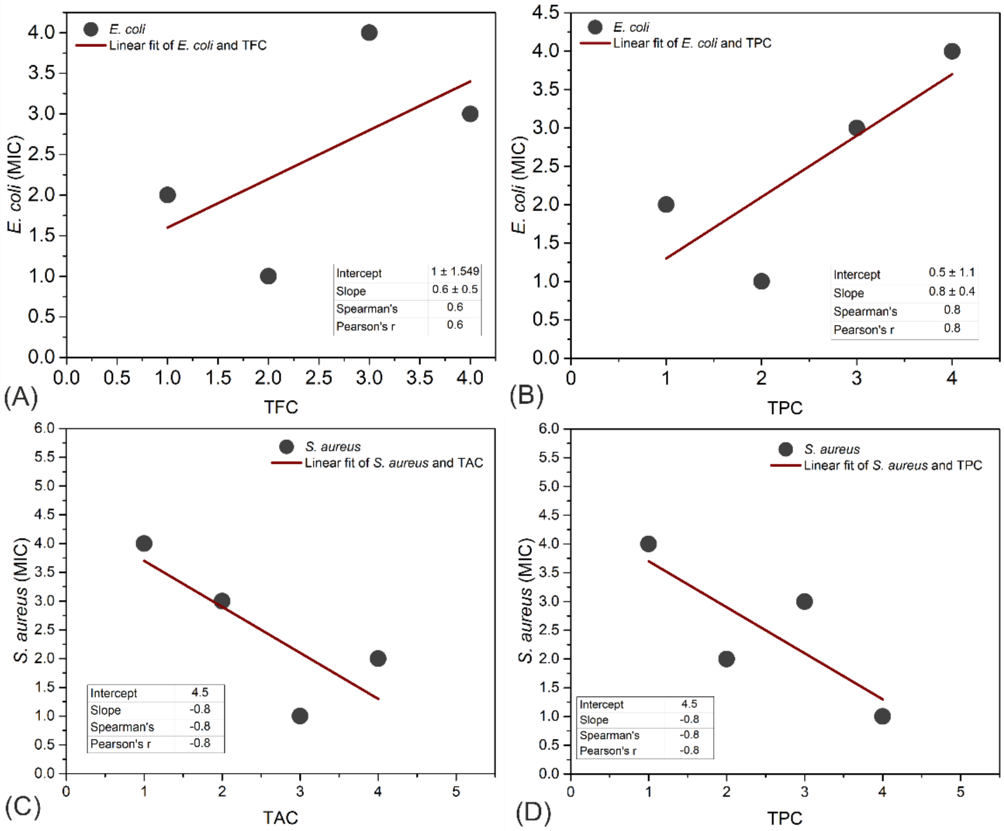

Figure S4. The total flavonoid (**A**) and polyphenol (**B**) content of the analyzed *Vinca* extracts is represented in relation to the ranked effects of the extracts against *E. coli*; The total alkaloid (**C**) and polyphenol (**D**) content represented in relation to the ranked effects of the extracts against *Staphylococcus aureus*.

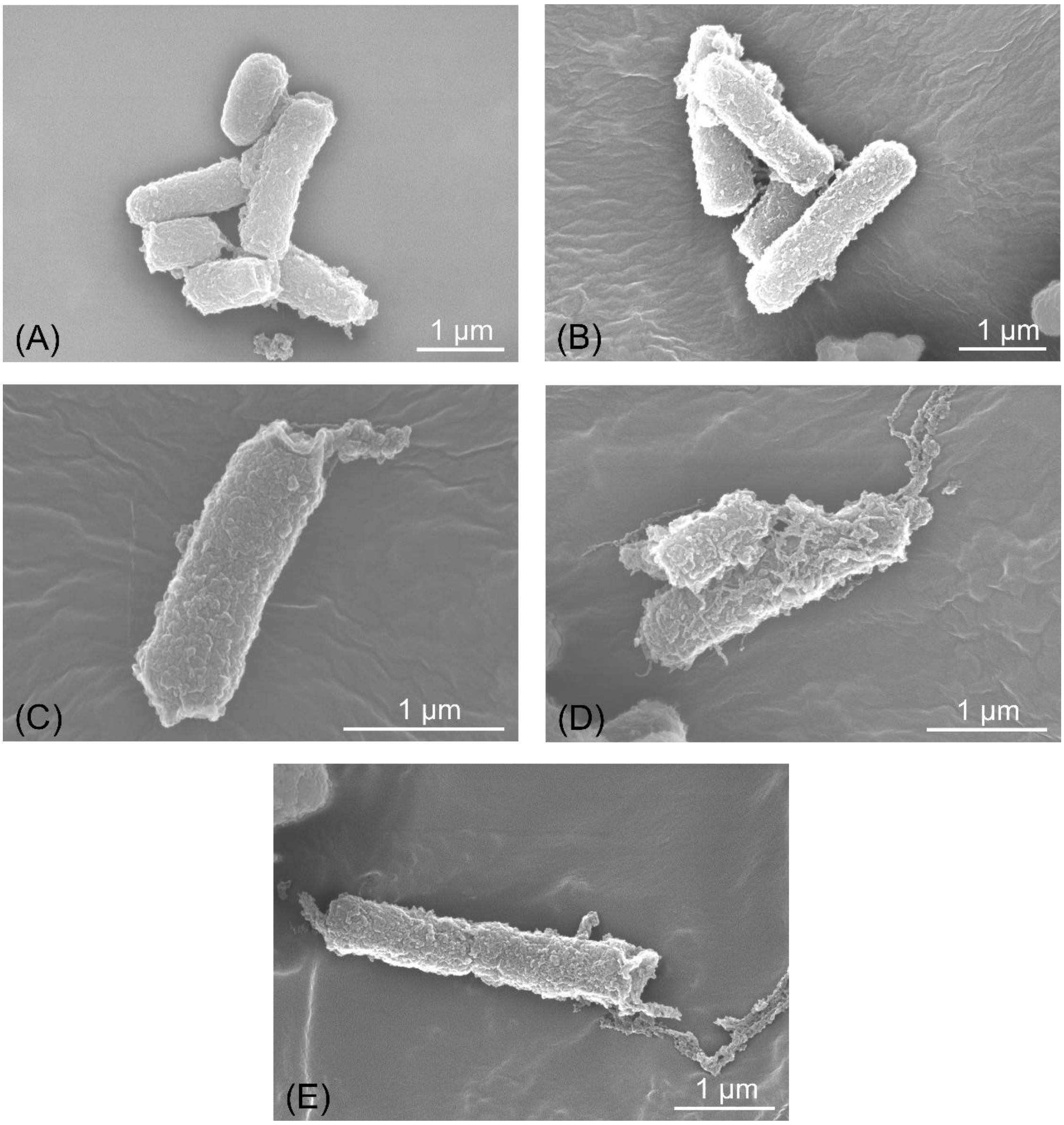

Figure S5. SEM micrographs of *E. coli* cells treated with *Vinca* leaf extracts at Minimal Inhibitory Concentration (MIC) values. (**A**) untreated control, treated with (**B**) *V. minor*, (**C**) *V. major*, (**D**) *V. major* var. *variegata*, (**E**) *V. herbacea*.

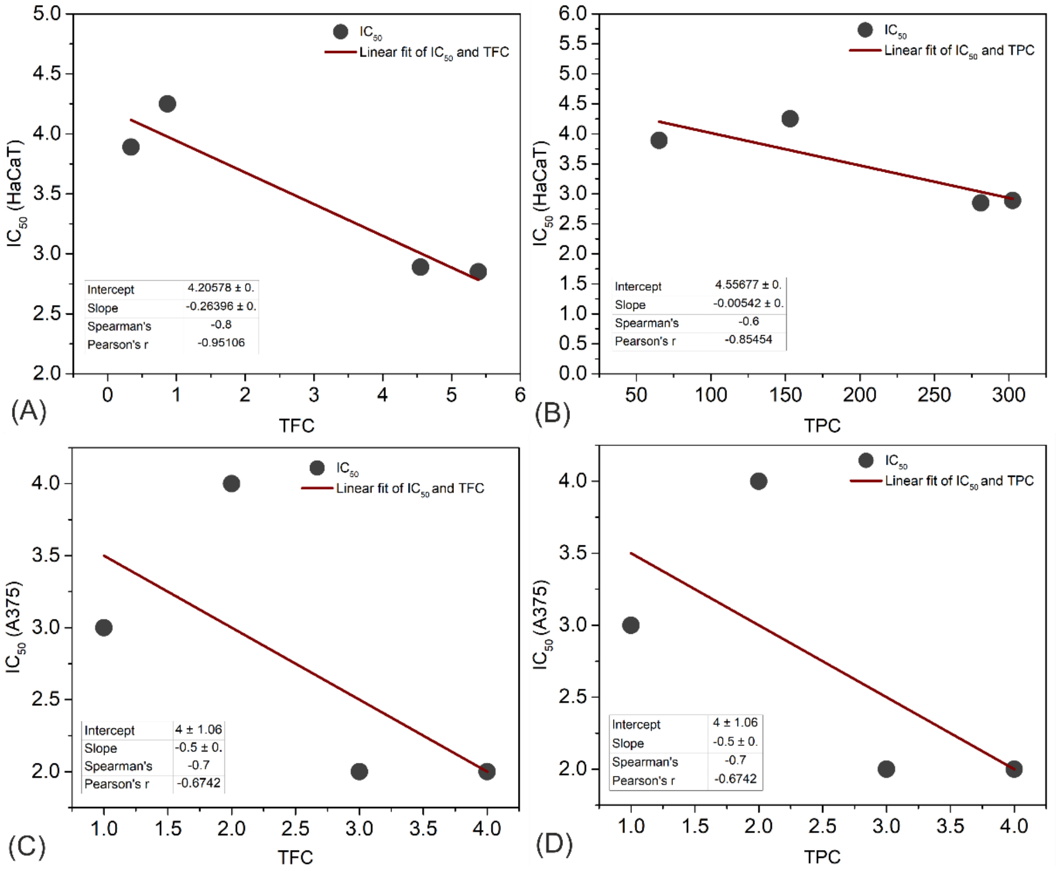

Figure S6. The total flavonoid (**A**) and polyphenol (**B**) content of the analyzed extracts represented in relation to the IC_{50} values observed against the HaCaT cells. The total flavonoid (**C**) and polyphenol (**D**) content of the analyzed extracts represented in relation to the IC_{50} values observed against the A375 cells.

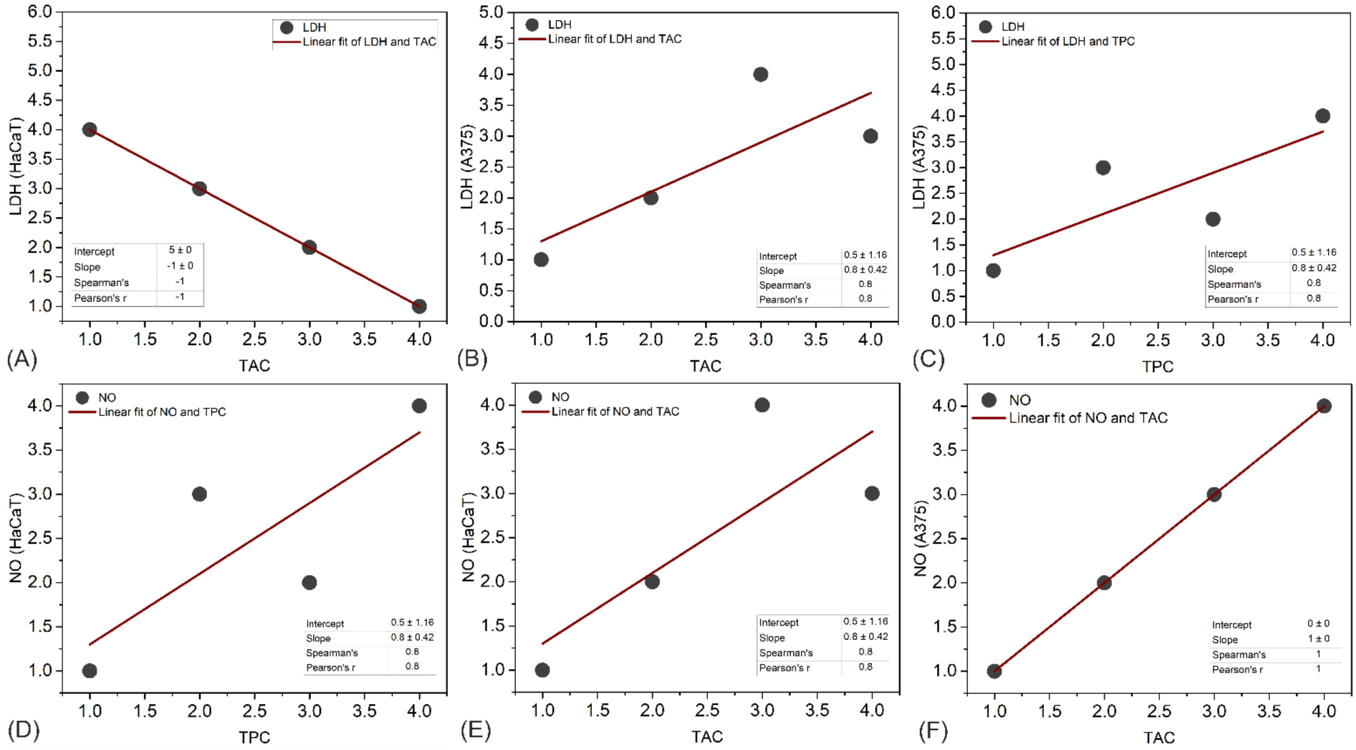

Figure S7. LDH release expressed in relation to the total alkaloid content in HaCaT cells (**A**) and A375 cells (**B**). The LDH release expressed in relation to the total polyphenol content in A375 cells (**C**). NO concentration expressed in

relation to the total polyphenol content (**D**) and total alkaloid content (**E**) in HaCaT cells. NO concentration expressed in relation to the total alkaloid content in A375 cells (**F**).

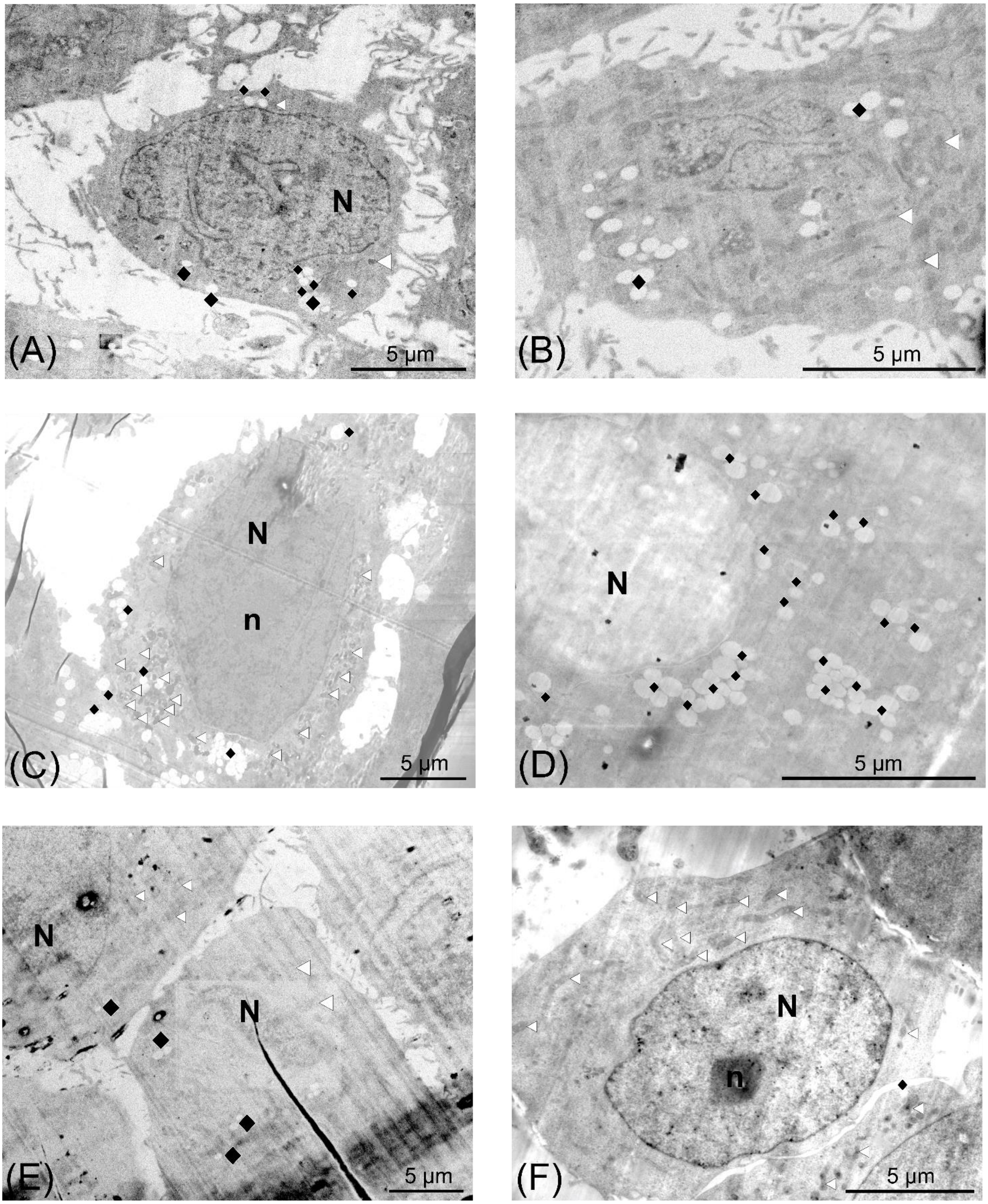

Figure S8. TEM micrographs of the HaCaT keratinocytes treated with *Vinca* extracts. (**A–B**) untreated control, treated with (**C**) *V. minor*, (**D**) *V. major*, (**E**) *V. major* var. *variegata*, and (**F**) *V. herbacea*. White triangles = mitochondria, black rhombs = vesicles, N = nucleus, n = nucleolus.

Table S1. Retention time, spectral features, phytochemical group, and chromatographic area (at 230 nm) for the peaks that were chemometrically analysed. Phytochemical group (Gr.) was attributed after PCA analysis based on spectral similarities. Standards were also included

Label	t_R (min)	Spectral Features (nm)	Gr.	Peak Area (%)	Label	t_R (min)	Spectral Features (nm)	Gr.	Peak Area (%)
Vm1	6.796	242, 310	Alk	6.36	VMv3	7.185	242, 310	Alk	2.29
Vm2	7.199	242, 310	Alk	7.25	VMv4	7.516	236	Alk	3.99
Vm3	7.516	236	Alk	8.85	VMv5	8.492	240	Bz	3.98
Vm4	8.940	234	Alk	10.90	VMv6	9.015	254, 354	Flv	3.72
Vm5	9.512	242, 326	Cin	13.76	VMv7	9.496	240, 326	Cin	12.94
Vm6	10.439	266, 346	Flv	1.23	VMv8	9.738	236, 326	Cin	3.40
Vm7	12.976	236	Alk	11.11	VMv9	9.823	266, 332	Flv	4.06
Vm8	14.455	244	Bz	1.42	VMv10	10.427	266, 346	Flv	1.78
Vh1	6.706	242, 310	Alk	5.59	VMv11	10.726	236	Alk	1.88
Vh2	7.113	242, 310	Alk	6.23	VMv12	12.799	266, 344	Flv	2.43
Vh3	7.456	236	Alk	11.35	VMv13	19.064	222, 275	Alk	2.54
Vh4	9.469	242, 326	Cin	7.65	VMv14	20.250	226, 275	Alk	1.66
Vh5	9.702	240, 326	Cin	3.47	VMv15	24.191	224, 294	Alk	1.99
Vh6	12.648	246, 316	Alk	8.71	dihydroxybenzoic	6.682	260, 294	Bz	-
Vh7	14.059	256, 354	Flv	9.33	chlorogenic	9.507	240, 326	Cin	-
Vh8	16.380	272, 300	Alk	3.01	hydroxybenzoic	9.721	256	Bz	-
Vh9	19.408	268, 290	Alk	2.44	caffeic	11.063	238, 324	Cin	-
VM1	6.984	242, 310	Bz	2.01	syringic	11.519	274	Bz	-
VM2	7.215	242, 310	Alk	7.65	rutin	14.090	256, 354	Flv	-
VM3	7.548	236	Bz	5.31	*p*-coumaric	14.260	226, 310	Cin	-
VM4	8.510	240, 372	Bz	5.82	isoquercitrin	14.893	256, 354	Flv	-
VM5	9.520	240, 326	Cin	6.05	ferulic	15.405	236, 322	Cin	-
VM6	10.724	238, 336	Flv	1.94	quercitrin	16.587	256, 350	Flv	-
VM7	13.041	238, 330	n.a.	1.92	myricetin	18.292	254, 372	Flv	-
VM8	14.677	222, 298	n.a.	1.02	berbamine	18.726	282	Alk	-
VM9	19.069	222, 275	Alk	2.70	vincamine	20.634	222, 270	Alk	-
VM10	20.249	226, 280	Alk	5.16	quercetin	22.426	254, 370	Flv	-
VM11	20.670	226, 286	Bz	1.86	kaempferol	30.058	264, 366	Flv	-
VM12	24.219	224, 294	Alk	3.64	vinblastine	31.744	216, 268	Alk	-
VMv1	5.760	242,310	Alk	1.00	galangin	35.272	310, 358	Flv	-
VMv2	6.974	236, 324	Cin	5.12					

*Vm = *V. minor*, VM = *V. major*, VMv = *V. major* var. *variegata*, Vh = *V. herbacea*, Alk = alkaloid, Flv = flavonoid, Bz = hydroxybenzoic acid, Cin = hydroxycinnamic acid, - = not detected.
